# The Institutional Worker

**Published:** 1918-08-31

**Authors:** 


### INSTITUTIONAL WORKER

**Being a Special Supplement to "The Hospital"**

### OUR BUREAU OF INFORMATION.

**Rules for Correspondents.**

1. Every letter must be accompanied by the coupon to be cut from the back cover (inside page) of THE HOSPITAL, current issue, and must contain the name and address of the correspondent with pseudonym for publication if desired. Replies by post cannot be given save under exceptional circumstances at the Editor's discretion.

2. Letters from Approved Homes in reply to special needs published in the Bureau should state terms and full particulars, and be sent prepaid under cover to the Editor of the Bureau with name written across coupon for identification.

3. Proprietors of Homes which have not yet been entered on the List of Approved Homes, but have spare accommodation likely to suit special needs, are invited to write for an application form for registration. The fee for registration, which includes two announcements of the Home in the Bureau and other privileges, is 10s.

4. All communications to be addressed to the Editor of THE HOSPITAL, 28 Southampton Street, Strand, London, W.C. 2, and marked "Bureau of Information."

### INSTITUTION FACTS AND FIGURES.

### Bedroom Suppers for Nurses Off Duty.

### Answer to July Question.

### By "Billee."

THE question was:

**In those hospitals having no kitchen in the Nurses' Home what arrangements can be made whereby the sisters and nurses who are off duty in the evening may have the comfort of supper in their bedrooms? What method may be adopted in order to avoid the dining-room maid being faced by a more or less depleted pantry when she wishes to lay breakfast? (The difficulty seems to be not so much as regards the food as the crockery.)**

At one of the larger London hospitals, where I was a former worker, the matter of bedroom suppers was never a very difficult one to arrange. With us, as in the case of your correspondent "Thorough," this privilege was, in the first place, only extended to sisters and staff nurses. It was the Home sister's duty to arrange that the members of the domestic staff should take it in turns one by one to take *supper duty*, her hours being usually from 7 to 12 P.M. Her duty was to take the trays to the rooms of those requiring supper; each nurse knew that it was expected of her, as soon as she had finished the repast, to place crockery and tray outside the door of her apartment. The maid on duty then collected the trays from each landing, washed up the dirty crockery, and saw that everything was in its place for the morning. To make up for the lateness of the hour, the duty was purposely made a short one, and the week on this special work was generally a favourite one with the maids attached to the Nurses' Home. It must be remembered that the nurses usually most require late supper in their own rooms on their off-duty time, and as they often return after attending the theatre, considerable latitude must be allowed, as regards the hours when suppers are served, if the privilege is to be a real boon to those concerned.

### Lectures on Infant Care.

### Elementary and Special Courses for Welfare Workers, Health Visitors, and Others.

A COURSE of elementary lectures intended for teachers, infant-welfare workers, mothers, etc., will be held at 1 Wimpole Street, W. (corner of Henrietta Street), on Mondays, from 5.30 to 6.30 P.M. from September 30 to December 16, 1918. The course is in preparation for the elementary certificate of the National Association for the Prevention of Infant Mortality, and all students who attend eight or more out of the twelve lectures may sit for examination. The fee for the course of lectures (there is no extra fee for the examination) is 5s., and for any one of the lectures 1s. All tickets must be applied for in advance. The first lecture, on September 30, will be by Dr. Truby King, C.M.G., on "The Hygiene of Infancy." The lecturers on successive Mondays will be: Miss Olive Hayden, Dr. Eric Pritchard, Miss Nora March, Dr. H. G. Adamson, Dr. Maurice Craig, Dr. H. C. Cameron, Capt. F. Vincent Denne, M.D., L.D.S., Dr. T. N. Kelynack, Mrs. H. B. Irving, Miss Chapman, Headmistress of the Page Green Girls' School, Tottenham, and Miss Pearson, First Superintendent, Holborn Maternity Centre.

NOTTINGHAM, SEPTEMBER 16 TO 20.

Special courses of lectures on Infant Care have also been arranged by the National Association for the Prevention of Infant Mortality and for the Welfare of Infancy. They are to be held at Nottingham and Swansea and are addressed to health visitors, nurses, midwives, school teachers, voluntary infant-welfare workers, committees of nursing associations, etc. The first course will be held in the Lecture Theatre of University College, Nottingham, from September 16 to 20 inclusive. Two lectures will be given daily, the fee for the course of ten lectures being 10s., or 1s. 6d. for a single lecture. It is hoped that arrangements will be made by the local health and education authorities for meeting the cost of fees, etc., of nurses and other workers under their jurisdiction, and those wishing to attend the course should therefore apply to the Medical Officer of Health or to the Secretary of the Education Committee for further information on the subject. The lecturers will be: September 16, Mrs. Scharlieb, C.B.E., M.D., and Sir Francis Champneys, Bart., M.D., September 17, Mrs. Sloan Chesser, M.B.; September 18, Dr. H. C. Cameron and Major G. H. Pooley, F.R.C.S.; September 19, Dr. E. J. Skinner and Miss Mildred Burgess, M.D.; September 20, Miss H. S. Newman and Miss Sarah Gray, F.R.C.S.

SWANSEA, OCTOBER 5 TO NOVEMBER 2.

A similar course will be held at the Secondary School for Boys, Dynevor Place, Swansea, on Saturdays, October 5, 12, 19, and 26, and on November 2. Two lectures will be given daily, from 11.30 A.M. to 12.30 P.M. and from 12.45 to 1.45 P.M., to allow those attending to be present also at the afternoon lectures organised by the National Council for Combating Venereal Disease. The fee for the whole course is 10s. 6d., 2s. 6d. for the two lectures on one day, or 1s. 6d. for a single lecture. Municipal workers desiring to attend and wishing to have their expenses paid are advised to make application to the Clerk to their own authority, several local authorities having intimated their intention of defraying the expenses of infant-welfare workers from their districts. The lecturers will be: October 5, Sir Francis

(*Continued on page 2, col. 3.*)

### AUGUST QUESTIONS.

### 1. War Vacancies.

**Give model "Terms of Appointment" applicable for the substitute of a hospital's administrative official who has been called to the Colours. Provision to be made for the possible return to duty of the official previous to the termination of the war and the filling of the appointment in case of the non-return of the official concerned. Describe fully any experiences in this connection that may prove of value to hospital managers generally.**

### 2. Bedroom Suppers for Nurses Off Duty.

**In those hospitals having no kitchen in the Nurses' Home, what arrangements can be made whereby the sisters and nurses who are off duty in the evening may have the comfort of supper in their bedrooms? What method may be adopted in order to avoid the dining-room maid being faced by a more or less depleted pantry when she wishes to lay breakfast? (The difficulty seems to be not so much as regards the food as the crockery.)**

#### RULES.

The following rules must be observed:—

1. Contributions must be written on one side of the paper. Brevity and terseness are desirable features. The MSS. must bear the name and address of the sender and be accompanied by coupon to be cut from the back cover (inside page) of the current issue of THE HOSPITAL. A pseudonym must be chosen if the name is not to be published.
2. Contributions must be addressed to the Editor of THE HOSPITAL, Institutional Worker Supplement, 28 & 29 Southampton Street, Strand, London, W.C. 2, should reach him before the end of the current month, and be marked in the left-hand corner "Facts and Figures."

**A minimum payment of Five Shillings will be made for each published answer.**

### QUESTIONS INVITED.

IN connection with our Question Box, we offer every month five shillings each for the two best questions which are sent in for consideration. The questions may be on any institutional subject and concern any department, from the secretary's to the porter's, or the matron's to the domestic staff; the one essential is that they must be practical and deal with points that have a definite relation to hospital or institutional administration, upkeep, finance, or management. Points relating to artificers' work, housekeeping, the laundry, out-patients, and other practical matters will be welcome. It is hoped that thus, when difficult or doubtful points arise in the course of their work, institutional workers will be encouraged to put them in the form of a question and send them to the Editor, THE HOSPITAL Bureau of Information, 28 & 29 Southampton Street, Strand, W.C. 2, marked "Question Box."

The best questions will be published in due course and our readers will be given the opportunity of answering them. Thus all institutional workers may be able to cooperate with the view of helping the work and smoothing the difficulties of each other. In short, we want the Question Box of the *Institutional Worker* to be freely used by every worker habitually.

### ENQUIRIES AND ANSWERS.

### *THE SICK AND IN NEED.*

#### Mental Hospital near Oxford.

PATIENTS are received at the Warneford Mental Hospital, Oxford, for a weekly charge of £2 2s. A certain number of patients are received for payments ranging from 5s. to 31s. 6d., the difference being made up from the endowment fund. Application should be made to the superintendent.—M. A. B.

#### Aid for Discharged Prisoner.

WE suggest that you make application to the Metropolitan Prisoners' Aid Society, 44 Burton Street, W.C. This Society gives assistance more especially to short-term male prisoners discharged from Pentonville Prison, and would, no doubt, be helpful to you in dealing with the case you refer to.—PENITENT.

#### School for Blind Woman.

WE suggest that you write, giving full particulars of the case, to the Principal and Secretary of the Royal School for the Indigent Blind, Leatherhead, Surrey. We believe they will train her in that branch of work which you suggest, providing she is a suitable case.—W. R. S.

### *EMPLOYMENT AND TRAINING.*

#### Post in Military Hospital in London.

WE cannot undertake to give you the names of military hospitals requiring nurses. There is a greater need than ever for trained nurses in London hospitals and elsewhere; provided you have the needful qualifications (you make no reference to your training) you should have no difficulty in getting an appointment through the ordinary channels, as, for instance, through the advertisement columns of the nursing papers and by application to the British Red Cross Society, 83 Pall Mall, S.W. 1. If you joined one of the Military Nursing Services you would have no guarantee of service in one particular place.—A. G.

#### Royal Sanitary Institute Courses of Lectures.

THE examinations of the Royal Sanitary Institute are officially recognised as qualifications for appointments by Government departments and municipal authorities. The courses of lectures given in London for sanitary officers commence September 16, for health visitors September 18, and for maternity and child-welfare workers September 23. Yes, there is a Centre in Sheffield. Write for application forms and full particulars to the Secretary, 90 Buckingham Palace Road, London, S.W. 1.

#### Post in Military Hospital or Nursing Home.

YOUR qualifications should readily secure you an appointment in a London hospital doing military work or in a good nursing home. See reply to A. G.—M. G. D.

### *MISCELLANEOUS.*

#### Old Rubber Gloves.

THERE are so many articles made of rubber in use in hospital requiring repair from time to time that it would be worth your while to keep all the old rubber gloves for this purpose. Apart from patching surgical gloves, the material is useful for mending nitrous-oxide bags and other similar apparatus, air cushions, and so on. As these gloves are usually made of pure rubber they can be dissolved in bi-sulphide of carbon for making rubber solution. Ask your dispenser about this. Inner tyres of bicycles can also be satisfactorily repaired with this rubber. In pre-war days most rubber manufacturing companies purchased old rubber, but they are not so ready to do it now. Most purchasers of old material will buy rubber. Write to your Borough Council.—NURSE.

#### Training for Health Visitor.

WE know of no hospital offering the training you require. Write to the Secretary, National Health Society, 53 Berners Street, Oxford Street, W.1, or the Secretary, Royal Sanitary Institute, 90 Buckingham Palace Road, London, S.W. 1.—E. M. W.

#### Book on Magnetism and Electricity.

"MAGNETISM and Electricity for Beginners," by H. E. Hadley, B.Sc., and published by Macmillan and Co., Ltd., St. Martin's Street, London, might be useful to you, or "Elementary Lessons in Electricity and Magnetism," by S. P. Thompson, D.Sc., F.R.S., published by the same firm. Both books are well illustrated.—STUDENT.

### LECTURES ON INFANT CARE.

(*Continued from page* 1.)

Champneys, Bart., M.D., and Miss Olive Hayden; October 12, Dr. Eric Pritchard; October 19, Mrs. Robert Hutchison, M.B.; October 26, Dr. H. W. Pooler; November 2, Mrs. Fowler, Superintendent of the Birmingham Women's Settlement School for Mothers.

A syllabus of the three courses and full particulars can be obtained from Miss J. Halford, Secretary, National Association for the Prevention of Infant Mortality and the Welfare of Infancy, 4 Tavistock Square, London, W.C. 1, to whom also early application should be made for tickets.

